# Functional data analysis to characterize disease patterns in frequent longitudinal data: application to bacterial vaginal microbiota patterns using weekly Nugent scores and identification of pattern-specific risk factors

**DOI:** 10.1186/s12874-023-02063-8

**Published:** 2023-10-26

**Authors:** Rahul Biswas, Marie Thoma, Xiangrong Kong

**Affiliations:** 1 University of Washington, Seattle, WA USA; 2 University of Maryland, College Park, MD USA; 3 Johns Hopkins University, Baltimore, MD USA

**Keywords:** Functional data clustering, Intra-person variability, Longitudinal data analysis, Unsupervised learning, Vaginal flora

## Abstract

**Background:**

Technology advancement has allowed more frequent monitoring of biomarkers. The resulting data structure entails more frequent follow-ups compared to traditional longitudinal studies where the number of follow-up is often small. Such data allow explorations of the role of intra-person variability in understanding disease etiology and characterizing disease processes. A specific example was to characterize pathogenesis of bacterial vaginosis (BV) using weekly vaginal microbiota Nugent assay scores collected over 2 years in post-menarcheeal women from Rakai, Uganda, and to identify risk factors for each vaginal microbiota pattern to inform epidemiological and etiological understanding of the pathogenesis of BV.

**Methods:**

We use a fully data-driven approach to characterize the longitudinal patters of vaginal microbiota by considering the densely sampled Nugent scores to be random functions over time and performing dimension reduction by functional principal components. Extending a current functional data clustering method, we use a hierarchical functional clustering framework considering multiple data features to help identify clinically meaningful patterns of vaginal microbiota fluctuations. Additionally, multinomial logistic regression was used to identify risk factors for each vaginal microbiota pattern to inform epidemiological and etiological understanding of the pathogenesis of BV.

**Results:**

Using weekly Nugent scores over 2 years of 211 sexually active and post-menarcheal women in Rakai, four patterns of vaginal microbiota variation were identified: persistent with a BV state (high Nugent scores), persistent with normal ranged Nugent scores, large fluctuation of Nugent scores which however are predominantly in the BV state; large fluctuation of Nugent scores but predominantly the scores are in the normal state. Higher Nugent score at the start of an interval, younger age group of less than 20 years, unprotected source for bathing water, a woman’s partner’s being not circumcised, use of injectable/Norplant hormonal contraceptives for family planning were associated with higher odds of persistent BV in women.

**Conclusion:**

The hierarchical functional data clustering method can be used for fully data driven unsupervised clustering of densely sampled longitudinal data to identify clinically informative clusters and risk-factors associated with each cluster.

## Background

Technology advancement has allowed more frequent monitoring of biomarkers to evaluate diseases or health conditions. For example, weekly measurements of grip strength collected during a 6-months period have been used to study how trajectories of muscle weakness served as a marker for adverse health outcomes in older adults [1]. Another scenario is studies using ecological momentary assessment technologies (EMA) where frequent data are captured to reflect peoples’ real-time behavior or emotion in their natural environments. Examples include studies using EMA data to identify patterns of illicit drug use behaviors [2, 3]. In our own collaboration in infectious disease epidemiology, a 2-year study in Uganda recorded weekly Nugent assay scores to assess the bacterial vaginosis (BV) status of women of reproductive age. The data structure resulting from all these studies entails more frequent follow-up sampling than traditional longitudinal studies where the number of follow-up is often small. Compared to cross-sectional or traditional longitudinal studies, the more frequent sampling offers a unique opportunity to study how intra-person variability contributes to disease etiology.

Traditional longitudinal data analysis methods such as mixed effects models or generalized estimating equation models focus on modeling the cross-sectional mean values and thus do not apply when the scientific goal is to explore and characterize the patterns of intra-person longitudinal changes. To analyze the frequently sampled data in the aforementioned BV dataset, we resorted to functional data analysis framework and developed a hierarchical functional clustering framework utilizing a set of data features by applying the functional non-parametric clustering method by Ferraty and Vieu (2006) [4].

BV is a common form of vaginitis in women and is related to various adverse health outcomes [5, 6]. BV can present with vague clinical manifestations like discharge, odor, and elevated vaginal pH (above 4.5). Up to $50\%$ of women with BV may not experience any symptoms [7, 8], and the causes and mechanisms underlying the condition are not well-understood [6]. Over a period of 2 years, a group of sexually experienced post-menarcheal women were monitored by the Rakai Health Sciences Program in Uganda, where the women collected their own vaginal samples on a weekly basis. The vaginal samples were scored on an integer scale from 0 to 10 using the Nugent criteria [9]. The goal with the densely sampled longitudinal data was to characterize fluctuations in vaginal microbiota and understand factors associated with persistence and resolution of BV in sexually experienced postmenarcheal women. It was hypothesized that the intra-person variability could be a clinical feature and encode different etiologic processes. The dataset was previously analyzed by dividing the Nugent scores into three groups based on the vaginal microbiota states: normal (Nugent score of 0-3), intermediate (Nugent score of 4-6), and BV (Nugent score of 7-10), and converting the frequently collected longitudinal data into the proportion of each of these three states over the entire follow-up period. Another analysis used conditional logistic regression to model the weekly transitional probabilities of the 3 states with relevant covariates [9–12]. Cheon et al. [13] developed a mixture Markov transition model formulation to allow identification of different covariates associated with different longitudinal transition probabilities. The transitions were between the 3 states determined by categorized Nugent scores, and the longitudinal patterns over time were pre-defined through visual inspection of the longitudinal trichotomized data.

Here we use a fully data-driven approach to characterize the longitudinal patterns of vaginal microbiota. The approach uses the original numerical values of Nugent scores and utilizes the intra-person variability to characterize the longitudinal patterns. More specifically, we applied functional data analysis (FDA) [14–17] based clustering methods in an unsupervised manner, and also extended Ferraty & Vieu’s functional clustering algorithm [4] by using additional data features during the clustering process. After the vaginal microbiota longitudinal patterns were identified, we then used multinomial logistic regression models to identify risk factors associated with each of the disease patterns.

The remaining of the paper is structured as below: the “The motivating example: a cohort study on vaginal microbiota changes in women from Rakai, Uganda” section provides an elaborate account of the aforementioned dataset that motivated this research; the “Review of functional principal components (FPC) [4]” - “Functional data clustering algorithm using FPC [4]” sections review Ferraty & Vieu’s functional data clustering algorithm using FPC [4]; and the “Extending the clustering algorithm to incorporate multiple data features” section describes our extended functional data clustering algorithm. The algorithm allows more usage of the features embedded in the longitudinal data and better differentiates patterns that may reflect distinct biological processes. “Results” section presents the results of the identified vaginal microbiota patterns for BV and the pattern specific risk factors using the motivating dataset. The “Discussion” section concludes the paper with a discussion.

## Methods

### The motivating example: a cohort study on vaginal microbiota changes in women from Rakai, Uganda

In the rural Rakai Region of Uganda, 312 consenting females between the ages of 13 and 39 participated in a two-year cohort study between 2001 and 2003. For up to two years, participants underwent weekly home-based self-collection of vaginal swabs for assessment of the vaginal microbiota and vaginal pH level. The self-collected vaginal swabs were placed on slides and allowed to air dry before being stained with Gram stain and scored using the Nugent quantitative morphologic categorization for vaginal microbiota, which yields integer scores ranging from 0 (normal) to 10 (BV). Detailed questionnaires on sexual risk behaviors and general health were administered at baseline and every 6 months. Every 6 months, a serologic sample was examined for the presence of HIV using HIV enzyme immunosorbent assays, with any discrepant findings being validated by Western blot.

**Fig. 1:**
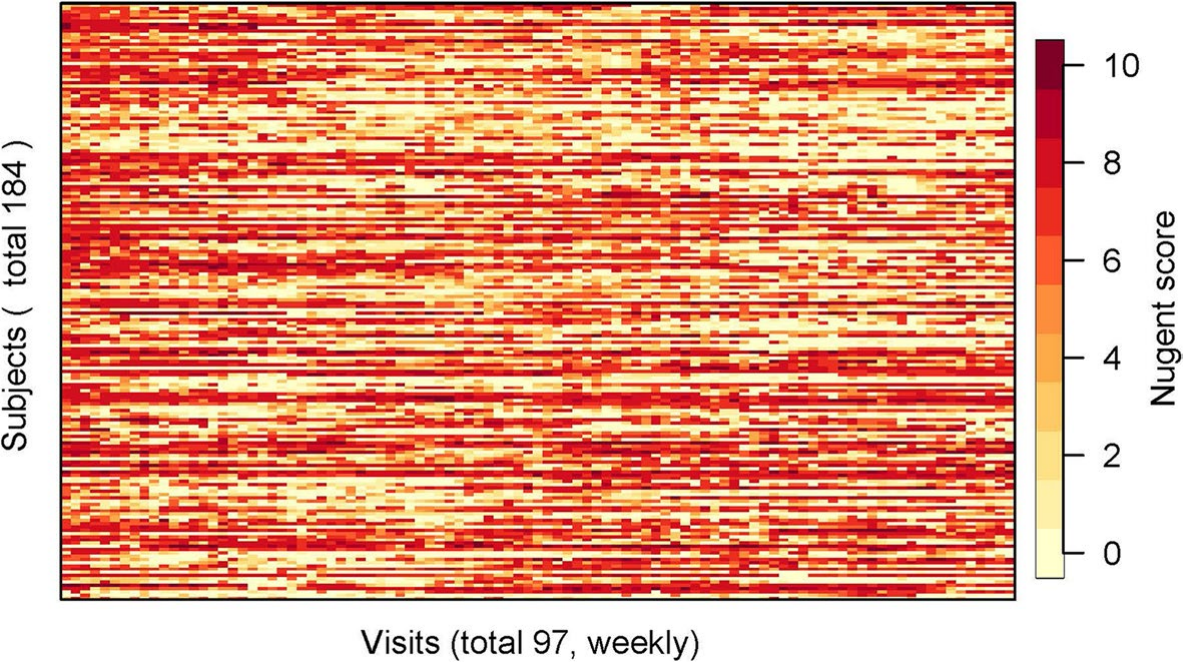
Nugent scores in Rakai dataset. This figure records Nugent scores for 97 weekly visits of each of 184 women who participated in at least 80% of weekly visits during two years. Remaining missing values have been imputed by linear interpolation. Each horizontal line corresponds to data from one woman. Each horizontal line is broken down into 97 colored intervals, where each interval indicates the Nugent score recorded in the corresponding weekly visit. Darker red indicates higher Nugent scores and more severe BV

The women who were sexually active, post-menarcheal, and who continued in the research for at least 18 months of observation were the focus of this analysis ($N=211$). Data of the subjects ($N=184$) who participated in at least 80% of the weekly visits during the two years were used for this analysis to characterize patterns of the longitudinal Nugent scores. Any remaining missing Nugent values were imputed by interpolating the immediately surrounding values if available and otherwise by carrying forward the previous non-missing value. The resulting Nugent scores observed for these 184 women over the 2 years are shown in Fig. 1. The potential risk factors and their baseline summary statistics are listed in Table 1 including covariates measured via the semi-annual questionnaires and HIV status.

**Table 1:** This table outlines the semi-annual covariates of interest that have been collected in the Rakai study

Covariates	Values	Baseline Summary/Frequency (% of 184)
Nugent score at start of interval ($BV_{0}$)	numeric value	Median: 6, Interquartile: 3-8, Range: 0-10 Percent of Nugent score in: The range of 0 to 3: 28.8% Intermediate range of 4 to 6: 23.4% BV range 7 to 10: 47.8%
HIV status with symptoms of AIDS	negative	165 (89.7%)
	positive without symptoms	15 (8.2%)
	positive with symptoms	2 (1.1%)
Age at baseline	$<20$ years	37 (20.1%)
	$\ge 20$ and $<25$ years	49 (26.6%)
	$\ge 25$ and $<30$ years	49 (26.6%)
	$\ge 30$ years	49 (26.6%)
Genital ulcer in past six months	yes	8 (4.3%)
	no	176 (95.7%)
Pregnancy test	pregnant	30 (16.3%)
	otherwise	154 (83.7%)
Source of bathing water	protected with well or tap or bore hole	35 (19.0%)
	partly protected being from uncovered wells	71 (38.6%)
	unprotected and from rains or ponds	78 (42.4%)
Current family planning use	birth control pills	14 (7.6%)
	Injection or implant hormonal contraceptives (Depo injection/Norplant)	28 (15.2%)
	none	142 (77.2%)
Sexual frequency	no sex	21 (11.4%)
(proportion of weekly visits that the woman reported	less than median value of frequency	78 (42.4%) -
sex in the last week over the past 6-months^a^)	greater than the median	85 (46.2%) -
Condom use in the past 6 months	never	118(64.1%)
	inconsistent	47 (25.5%)
	always used or no sex	19 (10.3%)
Partner’s circumcision status	circumcised	55 (29.9%)
	not circumcised	127 (71.1%)

^a^: the proportion of weekly visits with sexual activity was calculated among women who were sexually active in the past 6-months. The median was 65%

### Review of functional principal components [4]

Functional data analysis (FDA) extends the methods of multivariate statistics which concern $\mathbb {R}^d$ valued random variables to random variables taking values in function spaces [18]. In particular, the method of FPC reduces functional data to lower dimensions in an optimal way [19].

Let $\chi = \{\chi (t), t\in \mathcal {T}\}$ denotes a square integrable $L^{2}$-continuous stochastic process indexed over a compact interval $\mathcal {T}$. Denote the mean function as $\mu =E(\chi )$ and covariance function as $\nu (u,v)= cov \{\chi (u),\chi (v)\}$. Let $\phi _j, j\ge 1$ denote the eigenfunctions of *v* corresponding to eigenvalues $\lambda _j, j\ge 1$. The *j*-th FPC of $\chi$ is defined as

1 $$\begin{aligned} \xi _{j}=\int _{\tau }\left( \chi (t)-\mu (t)\right) \phi _{j}(t)\ dt \end{aligned}$$

According to the Karhunen–Loève expansion, it holds that $\chi (t)-\mu (t)=\sum _{j=1}^{\infty }\xi _{j}\phi _{j}(t)$, and the truncation with the first *q* terms $\sum _{j=1}^{q}\xi _{j}\phi _{j}(t)$ minimizes the $L_2$ distance between $\chi$ and any *q*-dimensional linear projection of $\chi$ [20]. Thus the first *q* FPC, $\xi _1,\ldots , \xi _q$ are an optimal dimension reduction of $\chi$ [21].

Let *i* index a study subject and $i=1,...,n$. Let ${x_{i}=(x_{i}(t_{1}),\ldots ,x_{i}(t_{K}))}$ be the observed discrete realization of $\chi$ for subject *i* and at recording times $t_1,\ldots , t_K$ where *K* is the number of time recordings for each *i* [22].

Let $\hat{\mu }(t_k)=\frac{1}{n}\sum _{i=1}^{n}x_{i}(t_k)$ be the estimated mean function at time $t_k$ and $w_{k}, k=1,\ldots , K$ denote the quadrature weights for the approximate integration [4, 23] of the integral in Eq. (1) over time $t_1,\ldots ,t_K$ and $W=diag(w_{1},\ldots ,w_{K})$. Let $\varvec{\hat{\phi }_{1}},\varvec{\hat{\phi }_{2}},\ldots$ be the orthonormal eigenvectors of the weighted covariance matrix of the observed data: $\frac{1}{n}\sum _{i=1}^{n}x_{i}'x_{i}W$, associated with its eigenvalues arranged in decreasing order. The estimate of $\phi _{j}(t_k)$, denoted by $\hat{\phi }_{j}(t_{k})$, is the *k*-th entry of $\varvec{\hat{\phi }_{j}},~k\in \{1,\ldots ,K\}$. Then, the integral in Eq. (1) is numerically calculated and estimated from observed data $x_i, i=1,\ldots , n$, as:

2 $$\begin{aligned} \hat{\xi }_{ij}=\sum \limits _{k=1}^{K}w_{k}(x_i(t_{k})-\hat{\mu }(t_{k}))\hat{\phi }_{j}(t_{k}) \end{aligned}$$

### Review of distance functions in function spaces [4]

Let $\chi _i$ and $\chi _{i'}$ be independent and identically distributed copies of the stochastic process $\chi$. The function,

3 $$\begin{aligned} d_q(\chi _i,\chi _{i'})=\sqrt{\sum \limits _{j=1}^{q}\left( \int [\chi _i(t)-\chi _{i'}(t)]\phi _{j}(t)\ dt\right) ^{2}} \end{aligned}$$

forms a semi-metric in the space of square-integrable stochastic processes for a fixed positive integer *q*.

For observed data ${x_i=(x_i(t_{1}),\ldots ,x_{i}(t_{K}))}$ and ${x_{i'}=(x_{i'}(t_{1}),\ldots ,x_{i'}(t_{K}))}$ at times $t_1,...,t_K$, $d_q(x_i,x_{i'})$ is estimated by,

4 $$\begin{aligned} \hat{d}_q(x_i,x_{i'})=\sqrt{\sum \limits _{j=1}^{q}\left( \sum \limits _{k=1}^{K}w_{k}(x_i(t_{k})-x_{i'}(t_{k}))\hat{\phi _j}(t_k)\right) ^{2}} \end{aligned}$$

The first *q* FPCs of $\chi _i$ and $\chi _{i'}$ can be denoted as $\xi _i=(\xi _{i1},\ldots ,\xi _{iq})$ and $\xi _{i'}=(\xi _{i'1},\ldots ,\xi _{i'q})$. Their estimates are denoted by $\hat{\xi }_i=(\hat{\xi }_{i1},\ldots ,\hat{\xi }_{iq})$ and $\hat{\xi }_{i'}=(\hat{\xi }_{i'1},\ldots ,\hat{\xi }_{i'q})$. Following Eqs. (1) and (2), note that,

5 $$\begin{aligned} d_q(\chi _i,\chi _{i'})=||\xi _i-\xi _{i'}||, ~~ \hat{d}_q(x_i,x_{i'}) = ||\hat{\xi _i}-\hat{\xi _{i'}}|| \end{aligned}$$

That is, the value of the semi-metric $d_q(\chi _i,\chi _{i'})$ between $\chi _i$ and $\chi _{i'}$ is identical to the Euclidean distance between the FPC vectors $\xi _i$ and $\xi _{i'}$ and likewise for their estimates.

### Functional data clustering algorithm using FPC [4]

Ferraty and Vieu [4] uses the proportions in small neighborhoods around the functional data points, defined using the semi-metric in Eq. (3) to hierarchically cluster the functional data. Let $S=\{\chi _{1},\ldots ,\chi _{n}\}$ denote a functional dataset with *n* subjects. Let $p_{i,h}=\frac{1}{n}\times card\{\chi _{i'} \in S:d_q(\chi _{i},\chi _{i'})<h\}$ denote the small neighborhood proportion of radius *h* around the functional data point $\chi _{i}$ for subject *i*, where *card* denotes the cardinality of a set. The $p_{i,h}$ is estimated from observed data by $\hat{p}_{i,h}=\frac{1}{n}\times card\{x_i':\hat{d}_q(x_{i},x_{i'})<h\}$ where *q* is the number of FPC used. The following summarizes the steps of the algorithm.

Step 1 Given a value of the neighborhood width *h*, evaluate the $\hat{p}_{i,h}$’s and estimate the density for $\{\hat{p}_{i,h}:i=1, 2, \ldots , n\}$ by standard density estimation methods [24]. Denote the density by $f_{h}$.
Step 2 Find the neighborhood width $\hat{h}$ that maximizes the entropy of $f_{h}$. For the next step, consider $f_{\hat{h}}$.
Step 3 Partition the $\hat{p}_{i,\hat{h}}$’s separated by the local minima of $f_{\hat{h}}$ to obtain the different classes. That is, $f_{\hat{h}}$ has *C* local minima at $m_{1},...,m_{C}$, then set the partitions to be $S_{1}=\{x_{i}:\ \hat{p}_{i,\hat{h}}\in (-\infty ,m_{1}]\},S_{2}=\{x_{i}:\hat{p}_{i,\hat{h}}\in (m_{1},m_{2}]\},...,S_{C}=\{x_{i}:\ \hat{p}_{i,\hat{h}}\in (m_{C},\infty )\}$ to be the different classes from *S*.
Step 4 Accept or reject the partition above based on this criterion: For any given sample denoted as *U*, define the heterogeneity index: $HI(U)=\frac{\hat{d}_q(M_{1,U},M_{2,U})}{\hat{d}_q(M_{1,U},0)+\hat{d}_q(M_{2,U},0)}$ where, $M_{1,U}$ and $M_{2,U}$ denotes the median, and the mode of samples in *S*, respectively. That is, *HI*(*U*) captures heterogeneity by the deviation of the median from the mode of the sample considered. Define the sub-sampled heterogeneity index, $SHI(U)=\frac{1}{B}\sum _{b=1}^{B}HI(U^{(b)})$, where $U^{(1)},...,U^{(B)}$ are *B* randomly generated subsamples of *U* (each subsample can be of half the size of *U* and is randomly drawn from *U* without replacement). That is, *SHI* is average *HI* over *B* random subsamples, making it a robust measure of heterogeneity of *U*. For the observed sample *S*, define the partitioning heterogeneity index $PHI(S;S_{1},...,S_{C})=\frac{1}{card(S)}\sum _{v=1}^{C}card(S_{v})\times SHI(S_{v})$. Define the splitting score $SC(S;S_{1},...,S_{C})=\frac{SHI(S)-PHI(S;S_{1},...,S_{C})}{SHI(S)}$. A decrease in the value of *PHI* or equivalently an increase in *SC* is desirable for the clustering because it increases intra-cluster homogeneity on an average over the clusters. For a given threshold, if the splitting score (*SC*) is above the threshold, partitioning is allowed, otherwise the parent sample is kept intact.
Step 5 If the partition is accepted in Step 4, then repeat Steps 1-4 using each of $S_1,\ldots ,S_C$ in place of *S* to further partition each of $S_1,\ldots ,S_C$.

The above method was applied on the Rakai dataset using the R code published by Ferraty and Vieu [4] using the default values of the tuning parameters and $q=2$ FPCs totally contributing to $\approx 66.45\%$ of the variance of the original data (the 3rd FPC only improved percentage of variance explained by less than $5\%$ and thus was not used). The tuning parameters were that the minimum sample size allowed for a cluster was 10; the set of small neighborhood width *h* for finding $\hat{h}$ was taken to be the set of values of $\hat{d}_q(x_i,x_{i'})$ in *S* and started with the least value; the *SHI* was calculated based on $B=1000$ randomly drawn subsamples, and the threshold for the splitting score *SC* was 0.05.

However, the functional clustering algorithm only uses one data feature for hierarchical clustering: the small neighborhood proportion in a neighborhood around each functional data point. This feature essentially uses the distance information as measured by the semi-metric of functional data points and thus encodes intra-person variability. On the other hand, other features may also be informative, for example, the estimated values of the principal components of the data points themselves. This motivated the expanded clustering algorithm below.

### Extending the clustering algorithm to incorporate multiple data features

Ferraty and Vieu’s methodology separates clusters by the minima of the density for the feature of small neighborhood proportion ($f_{\hat{h}}$) and using this feature to cluster hierarchically. Conceptually the algorithm can be extended by considering the density of other features such as the values of the FPC for each hierarchy of clustering.

**Figure:**
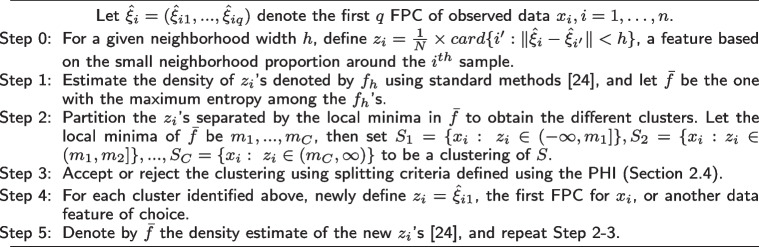
**Algorithm 1** Adapted functional clustering algorithm over multiple features

The extended clustering algorithm is provided in Algorithm 1. Steps 0-3 remain the same as the steps in Ferraty and Vieu’s original algorithm (see “Functional data clustering algorithm using FPC [4]” section) and use the small neighborhood proportions calculated based on the distances of the functional data for clustering. In Step 4, a new data feature, the first FPC for a subject, is used to further partition each cluster resulted from Step 3. The first FPC captures the greatest variance, followed by the second principal component and so on. Therefore, the clustering can be conducted in a hierarchical manner while utilizing multiple data features. Statistically, whether a data feature is relevant in clustering can be assessed by whether using it can further partition the sample based on the splitting score (*SC*) criterion: if the *SC* is less than the cutoff value, using this data feature will not lead to further partitioning of the the clusters already obtained by using the previous feature(s).

### Identifying risk factors after defining the patterns (clusters) using GEE

Exploratory analysis was further conducted to identify risk factors associated with each identified cluster. Since the exposure variables were measured every 6 months in the Rakai dataset (Table 1), we first split the two-year Nugent score time series of each subject into 6-months semiannual intervals. The Nugent score patterns over 6-months intervals were classified using Algorithm 1. Subsequently, generalized logistic regression modeling was used to model the semiannual class memberships as a function of the corresponding semiannually collected covariates, including age at the study baseline, HIV status at the beginning of the interval, and health status and sexual behaviors reported in the survey at the end of the interval (because the recall period was the past 6 months, e.g. whether there was genital ulcer in the past 6 months Table 1). The generalised estimating equation method [25] was used to account for the correlation within an individual due to the multiple semiannual intervals.

The generalized logistic regression is $log(\frac{\pi _{ilc}}{\pi _{ilC}})=\beta _{0c}+\beta _{c}'x_{il}$, where $Y_{il}$ is the class membership identified from the above clustering process for subject *i*, $(i = 1,...,N)$ at semiannual interval *l* $(l = 1, 2, 3, 4)$, $x_{il}$ is the covariates vector for subject *i* at interval *l*, and $\pi _{itc} =P(Y_{il} = c)$, $c={1, 2, . . . , C}$ with *C* being the number of classes determined from the clustering process. The parameters were estimated by Generalized Estimating Equations (GEE) method, using SAS GENMOD Procedure.

## Results

### Patterns identified only using the feature of small neighborhood proportions for clustering

**Fig. 2:**
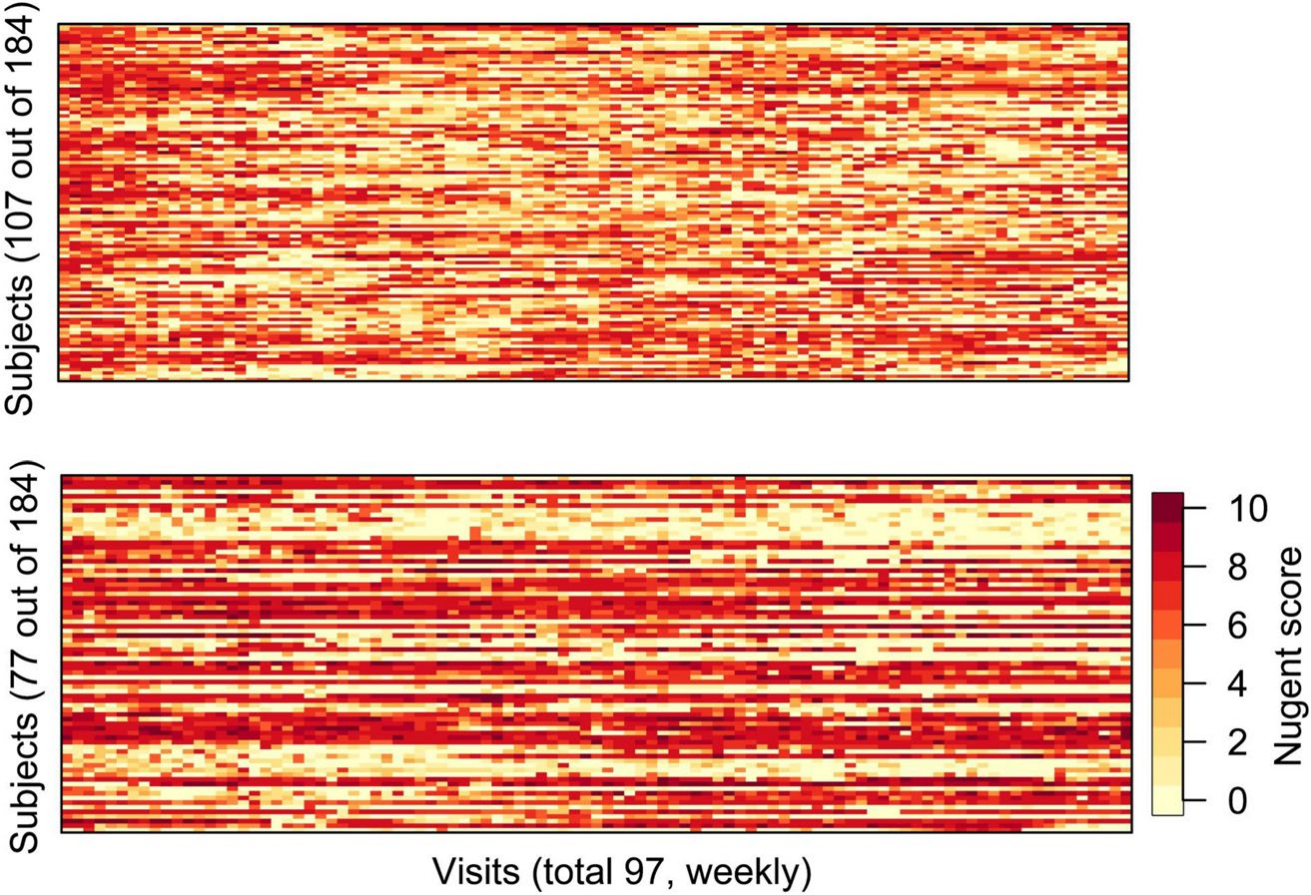
Clustering results of Ferraty & Vieu’s FDA method. This figure shows clusters obtained by Ferraty & Vieu’s FDA clustering method described in the “Functional data clustering algorithm using FPC [4]” section. Only the feature of small ball proportions was used for clustering, resulting in 2 clusters. Cluster 1 (top panel) suggests more fluctuations of Nugent scores over time, and Cluster 2 suggests more stable Nugent scores. However, Cluster 2 (bottom panel) does not differentiate women with stable high Nugent scores (i.e. with BV) versus women with low Nugent scores (i.e. no BV) which clearly are two distinct biological states

We first applied Ferraty & Vieu’s clustering method on the Nugent score time-series which spanned a 2-years of period with 184 subjects (see “Methods” section). The clustering yielded 2 classes (See Fig. 2) with a *SC* of 0.1723. This is equivalent to using Algorithm 1 with only the feature of small neighborhood proportions . The resulting 2 classes are largely distinguished by their persistence (or lack of) in the magnitude of Nugent scores. The first identified class indicates large fluctuations of the Nugent scores, suggesting a lower persistence in either the normal or BV states; whereas the second identified class indicates higher persistence in one of the normal or BV state. Utilizing only the small neighborhood proportion to cluster seemed to result in an overly crude clustering of BV longitudinal scores: as seen in Fig. 2, cluster 2 actually contains women who had consistently high Nugent scores indicating a disease state, as well as women who had consistently low Nugent scores indicating a disease free state. These two groups of women clearly belonged to distinct BV related disease processes, but they were not separated by using only the small neighborhood proportion to cluster the data.

### Patterns identified using the extended clustering algorithm

**Fig. 3:**
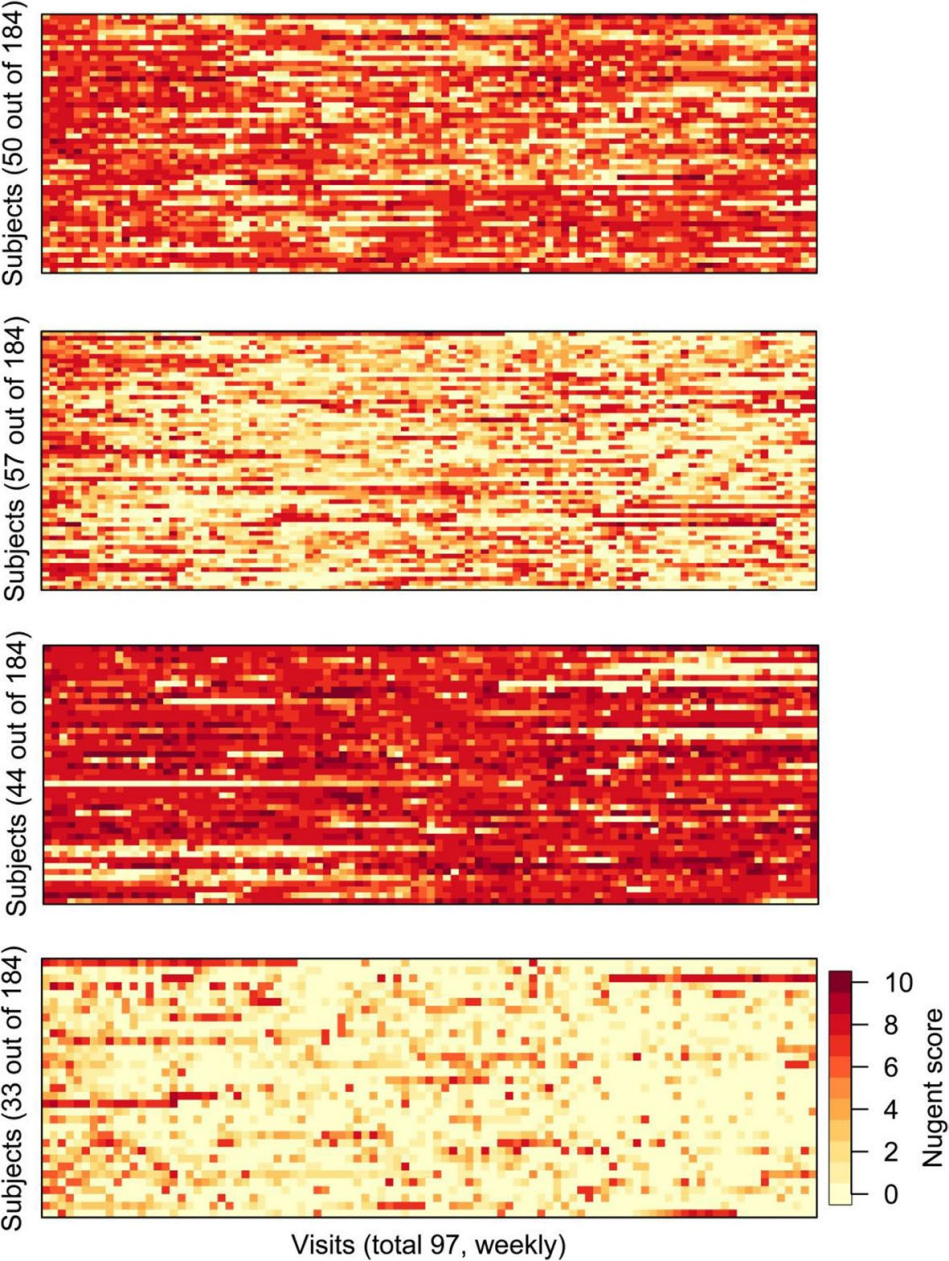
Clustering results of Algorithm 1. These figures show the four classes (Class 1 to Class 4: top to bottom panels) obtained by our extended FDA clustering method (Algorithm 1) described in the “Functional data clustering algorithm using FPC [4]” section using the weekly Nugent scores over 2 years. Two data features were used: the small neighborhood proportions as in Ferraty & Vieu’s method and the first FPC

Using the features of both the small neighborhood proportions and also the values of the first FPC, applying Algorithm 1 further bifurcates each of the 2 classes obtained in “Patterns identified only using the feature of small neighborhood proportions for clustering” section, yielding 4 classes in total (Fig. 3). The *SC* of the resulting 4 classes is 0.4011, larger than the *SC* of 0.1723 for the 2 classes when only using the small neighborhood proportions (see the “Patterns identified only using the feature of small neighborhood proportions for clustering” section). A greater *SC* indicates lower intra-cluster heterogeneity and thus is desirable.

In Fig. 3, data of Class 1 demonstrate large fluctuation of Nugent scores which however are predominantly in the BV state (high Nugent scores); data of Class 2 also demonstrate large fluctuation of Nugent scores but predominantly the scores are in the normal state; data of Class 3 show a pattern of persistent BV state; and data of Class 4 show a pattern of persistent normal ranged Nugent scores. These classes represent distinct biological risks to BV, representing women who had a lower risk of developing the disease (persistent low Nugent scores), women who had persistence of BV (persistently high Nugent scores), and those whose vaginal microbiota states fluctuated during the 2 years of follow-up.

### Pattern specific risk factors

**Fig. 4:**
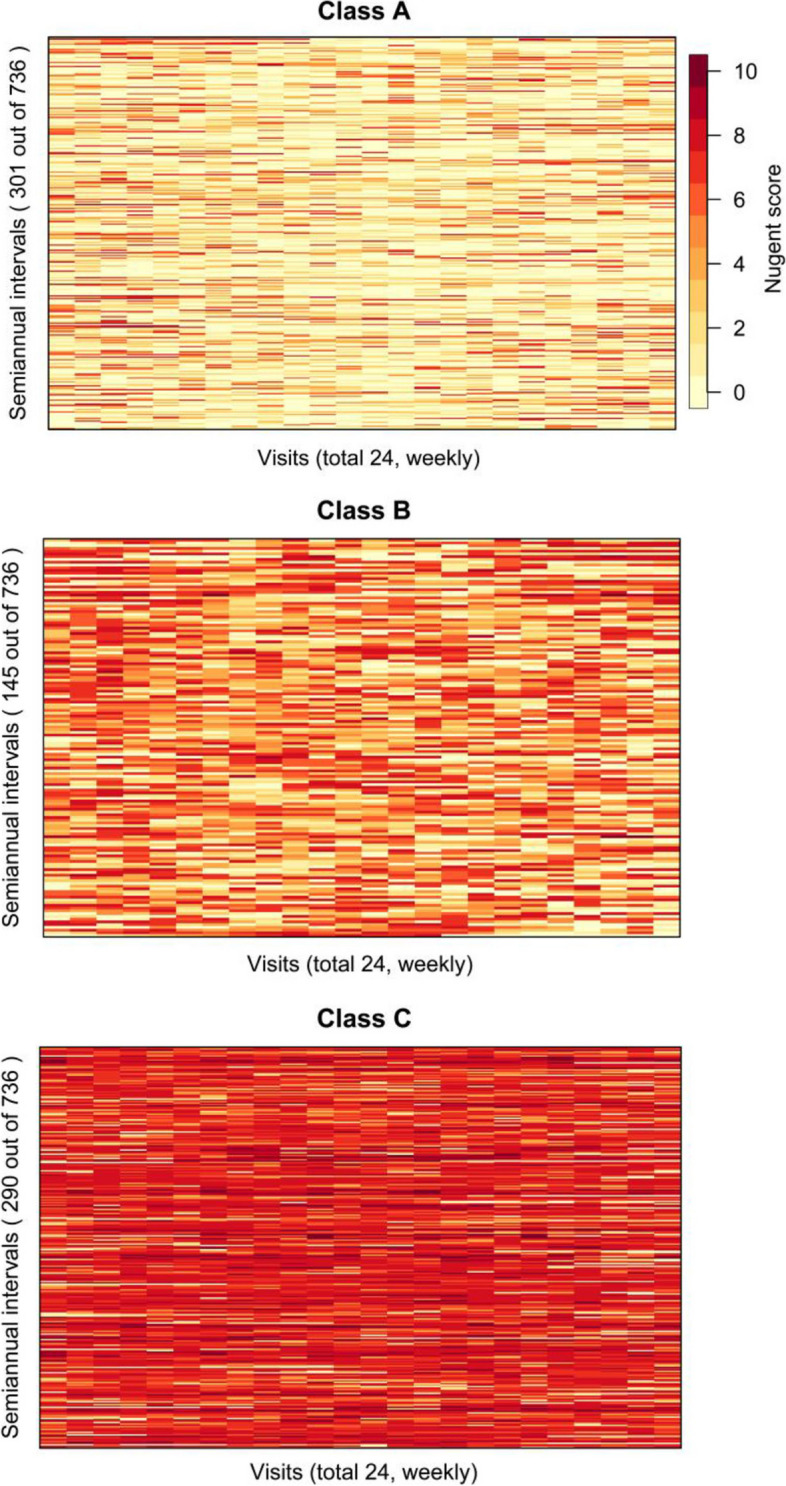
Clusters in Nugent score semi-annual intervals. Three classes were identified using Nugent scores in semiannual intervals.The extended method (Algorithm 1) was applied. Class A shows a pattern of persistent normal ranged Nugent score; Class B indicates a pattern of fluctuating Nugent scores; and Class C indicates a pattern of persistent BV state, i.e. high Nugent scores

Algorithm 1 was applied on the semi-annual Nugent score series to classify Nugent score patterns over 6 months. The identified patterns are shown in Fig. 4. The clustering results were similar to what was found earlier using the 2-year Nugent scores (Fig. 3), except that 3 clusters (patterns) were identified due to a reduction of variability over time associated with the semi-annual intervals as compared to two years. The clusters constitute women with persistently low Nugent scores ( Fig. 4, Class A), women with persistently high Nugent scores indicating a persistent BV state (Fig. 4, Class C), and women whose Nugent scores fluctuated between low and high Nugent scores (Fig. 4, Class B). The *SC* of the clustering is 0.0950.

When Ferraty & Vieu’s algorithm with only the feature of small neighborhood proportions was used to cluster the semi-annual data, the *SC* of clustering was 0.0606, and similar to the clustering result when using the algorithm on the 2-year data, 2 clusters were identified with one cluster being of women with fluctuating Nugent scores (i.e Class B) and the other cluster being of women with stable Nugent scores. Applying Algorithm 1 and incorporating the first principal component as the second data feature bifurcated the latter cluster into two clusters (Class C composed of women with stable high scores versus Class A composed of women with stable low scores), and increased the *SC* to 0.0950.

In the multinomial logistic regression model, we used the class with persistently low Nugent scores, Class A, as the reference group. The estimated odds ratios (OR) and their 95% confidence intervals based on the sandwich estimates for the standard errors and the *p*-values are presented in Table 2 for comparing the class of women with fluctuating Nuegent scores with the reference group and in Table 3 for comparing the class of women with persistently high Nugent scores with the reference group.

**Table 2:** Risk factors associated with Class B (fluctuating) compared to Class A (normal) using Multinomial Logistic GEE Regression

Covariates	Comparisons	Odds Ratio	95% CI	*p*-value
Nugent score at start of interval ($BV_{0}$)	Every unit increase in Nugent score	1.26	[1.17,1.34]	<0.0001
**Indicator Variables**				
HIV/AIDS status	HIV+ with no symptoms vs. HIV-	1.56	[0.52,4.67]	0.43
	HIV+ with symptoms vs. HIV-	0.32	[0.08,1.39]	0.13
Age at baseline	$(\ge 20, <25)$ vs. $<20$	0.37	[0.18,0.76]	0.007
	$(\ge 25, < 30)$ vs. $<20$	0.43	[0.21,0.90]	0.025
	$\ge 30$ vs. $<20$	0.59	[0.28,1.24]	0.16
Genital ulcer in past six months	yes vs. no	1.08	[0.31,3.79]	0.90
Pregnancy test	pregnant vs. not pregnant	0.58	[0.27,1.26]	0.17
Source of bathing water	partially protected vs. protected	0.74	[0.39,1.41]	0.36
	unprotected vs. protected	0.841	[0.43, 1.64]	0.61
Current family planning use	birth control pills vs. none	1.16	[0.49,2.78]	0.74
	Injectable/Norplant vs. none	1.20	[0.68,2.13]	0.53
Sexual frequency	< median vs. no sex	1.25	[0.53,2.94]	0.61
	> median vs. no sex	1.376	[0.56,3.39]	0.49
Condom use	inconsistent vs. always use or no sex	1.51	[0.84, 2.71]	0.17
	never used vs. always use or no sex	0.99	[0.37, 2.66]	0.99
Partner’s circumcision status	circumcised vs. not	0.79	[0.48,1.29]	0.34

**Table 3:** Risk factors associated with Class C (persistent BV) compared to Class A (normal) using Multinomial Logistic GEE Regression

Covariates	Comparisons	Odds Ratio	95% CI	*p*-value
Nugent score at start of interval ($BV_{0}$)	Every unit increase in Nugent score	1.68	[1.56,1.80]	<0.0001
**Indicator Variables**				
HIV/AIDS status	HIV+ with no symptoms vs. HIV-	1.69	[0.53,5.38]	0.38
	HIV+ with symptoms vs. HIV-	0.73	[0.22,2.43]	0.61
Age at baseline	$(\ge 20, <25)$ vs. $<20$	0.41	[0.17,1.01]	0.05
	$(\ge 25, < 30)$ vs. $<20$	0.47	[0.21,1.09]	0.08
	$\ge 30$ vs. $<20$	0.52	[0.23,1.18]	0.12
Genital ulcer in past six months	yes vs. no	0.51	[0.16,1.66]	0.27
Pregnancy test o	pregnant vs. not pregnant	1.33	[0.65,2.72]	0.44
Source of bathing water	partially protected vs. protected	1.28	[0.57,2.91]	0.54
	unprotected vs. protected	2.08	[0.95, 4.56]	0.07
Current family planning use	birth control pills vs. none	0.81	[0.27,2.40]	0.70
	Injectable/Norplant vs. none	1.76	[0.95,3.24]	0.07
Sexual frequency	< median vs. no sex	1.29	[0.59,2.82]	0.53
	> median vs. no sex	1.19	[0.52,2.76]	0.68
Condom use	inconsistent vs. always use or no sex	1.53	[0.80, 2.91]	0.20
	never used vs. always use or no sex	1.31	[0.46, 3.73]	0.61
Partner’s circumcision status	circumcised vs. not	0.62	[0.37,1.04]	0.07

## Discussion

As expected, higher Nugent score at the start of an interval was significantly associated with higher odds of vaginal microbiota fluctuation (Table 2) and BV persistence (Table 3). Younger age group of $<20$ years was significantly associated with higher odds of vaginal microbiota fluctuation and also higher odds of persistent BV. Unprotected source for bathing water such as rains or ponds compared to protected source had a doubled odds of persistence in BV microbiota (95%CI: 0.95 to 4.56, *P*-value 0.07). These findings are consistent with those previously reported for this cohort [12]. In the current analysis, a woman’s partner’s being circumcised also was associated with lower odds of BV persistence in the woman (OR= 0.62, 95%CI 0.37-1.04, *P*-value 0.07). This finding conforms to the knowledge that male circumcision reduces the prevalence of BV in the female partners’ by 40% concluded from a randomized controlled trial of male circumcision from Uganda.

Use of injectable/Norplant hormonal contraceptives (mainly injectable depot medroxyprogesterone acetate [DMPA] in Rakai) for family planning increased the odds of persistent BV in women (OR=1.76, 95% CI 0.95-3.24, *P*-value 0.07). In a previous analysis of this dataset [12], such hormonal contraception use was not detected as a potential risk factor for BV chronicity. This previous analysis defined the outcome of “BV chronicity” as the proportion of weekly Nugent scores that fell into the BV category (i.e. Nugent score $>7$) during the 6-months intervals. This approach condensed the original series of weekly Nugent scores of a woman into a scalar measure and thus was not an efficient and optimal use of the data (this shortcoming was indeed the motivation of this current study). Additionally, the scalar measure could not reflect the dynamics of vaginal microbiota or the intra-person variability over time. In contrast, the current analysis used a data-driven approach to characterise the patterns of longitudinal Nugent scores and identified the association of injectable/implant hormonal contraceptive use with a persistent BV state. Injectable DMPA use has been reported to be associated with increased risk of HIV acquisition [26]. The identified biological mechanisms from *in-vitro* studies include DMPA’s effect on microbiota and genital tract barrier function and tissue architecture [27]. Clinical studies using 16S rRNA gene sequencing or quantitative polymerase chain reaction have also reported DMPA use altered vaginal microbiota in black women [28, 29]. Our current identified association between DMPA use with BV chronicity benefited from the data-driven approach that used the raw Nugent scores and the intra-person variability in the data.

These findings suggest that risk factors for shifts in the vaginal microbiota are multifactorial and potentially include factors that increase biologic susceptibility, environmental exposures, and partner characteristics. Some factors may be modifiable and may lead to strategies for prevention and care of BV. Further studies are needed to elucidate the mechanism of how these risk factors may influence vaginal microbiota fluctuation and persistence of BV.

In prior analyses of this dataset, the raw Nugent scores were condensed into 3 categories (0-3 Normal; 4-6 Intermediate; and 7-10 BV). Some studies summarized the longitudinal data into cross-sectional proportions of each category. This provided a summary measure of BV disease burden but prohibited the exploration of biological knowledge embedded in the intra-person variability in the Nugent scores and vaginal microbiota [9, 10]. Another study [13] prespecified 3 subgroups based on visual inspection of the 2-year trichotomized Nugent score categories: the first group consisted of women who fluctuated between normal and intermediate states during the 2 years; the first group consisted of women who persisted with intermediate vaginal microbiota state and BV; and the first group were women who transitioned across all 3 states. The clustering method presented here used the actual Nugent scores (0-10) in a data driven manner. This fuller use of the original numerical values further differentiated the group of women who transitioned across all three states into those who generally had lower Nugent scores from those who transitioned across all three states but more often stayed with high Nugent score over the 2 years (Fig. 3). The latter subgroup may be associated with increased risk of other adverse outcomes.

Prior epidemiological studies of BV primarily relied on measurements of samples collected at one or a few time points every a few months apart [30, 31]. Several recent studies used daily or twice weekly sampled swabs from healthy women or women of high risk of BV over a 10 to 16 weeks period to characterize vaginal microbes and transition dynamics of bacterial species in vaginal microbiota [32, 33]. The Rakai BV study enrolled a relatively large cohort of women and collected vaginal samples weekly for 2-years, allowing explorations of the intra-person variability in vaginal microbiota and its role in the etiologic pathways of BV. But this study has important limitations. First, the covariates were measured semi-annually whereas Nugent scores were measured weekly. We assessed the associations of the covariates with BV patterns in the corresponding semi-annual intervals. More frequent such as weekly observations of covariates may allow a better understanding of the associations of hygienic and sex behavioral factors with BV status in women. Second, Nugent score does not provide information on the bacterial composition in vaginal microbiota. Thus our analysis cannot provide insight on the potential role of changes in bacterial species on women’s BV status nor how hygienic and sex behavioral factors may influence the bacterial composition in vaginal microbiota. Studies involving profiling of bacterial composition of densely sampled vaginal samples have shown that the composition of bacterial species may change and the temporal dynamics of the microbiota was correlated with clinical BV stage [32, 33]. Another study used Markov transition models on quarterly sampled data of vaginal bacteria communities and identified specific bacteria species that may be be targeted by interventional strategies to improve bacteria-associated reproductive health [31].

Frequently sampled longitudinal data have become increasingly available in recent years. Such data greatly expand the information from a single or a few time points of measurements of biomarkers and allow researchers to explore the clinical utility of the whole process of fluctuations of biomarkers in reflecting a disease or health condition. We applied FDA methods to identify vaginal microbiota patterns using the Nugent scores. Compared to traditional longitudinal data methods, FDA is known to perform better in higher dimensions [34] and can be applied when data are collected at different intervals for different subjects [19]. In particular, building on Ferraty & Vieu’s functional clustering algorithm, our extended algorithm utilize more data features in the clustering. This resulted in a classification of the Nugent score processes with improved clinical interpretability. Additionally, the classes were identified in a data driven manner using the raw longitudinal Nugent scores and reflected homogeneous subgroups of women that shared similar intra-person variability patterns.

The original Ferraty & Vieu’s method uses the small ball proportions to cluster the functional data. Considering that the proportions have removed information of the values themselves of the functional data, we extended Ferraty & Vieu’s algorithm by further clustering the functional data using other data features, such as the estimated first FPC itself for each subject. The extended algorithm provides a flexible framework of unsupervised learning of frequently sampled longitudinal data to identify biologically or clinically meaningful patterns. The data features to be used in the extended algorithm are chosen by the user, and the number of features to consider should depend on the problem at hand. Using a large number of data features for clustering may not be accepted by the splitting score criterion, or the resulting clusters may not be clinically or biologically interpretable. Our extended algorithm uses a one-feature-at-a-time hierarchical approach instead of clustering using a set of features simultaneously. The latter may be over fitting the data and results in spurious clusters in the presence of many features. With the hierarchical approach, the extended clustering algorithm checks one feature at a time and determines whether the feature is informative for the clustering before proceeding to the next one. This allows informative features to be used in clustering and also allows users’ control of data features to ensure that the non-supervised learning of data can generate scientifically interpretable subpopulations. For example, if the frequently sampled longitudinal biomarker measurements pertain to a degenerative process, then the data feature may include a measure of the rate of decline of the biomarker measurements to inform the clustering process.

The clustering methods may also be adapted to characterize disease processes using multivariate frequently sampled longitudinal data. For example, it may be applied to frequenly sampled vaginal microbiota data obtained from higher resolution tools using 16S rRNA gene amplicon sequencing to explore species-specific longitudinal patterns, which may allow identifications of specific pathogenic vaginal organisms with persistent or fluctuating patterns. The original and extended clustering methods are model-free and do not require parametric distribution assumptions. Thus they can be applied to a wide variety of high dimensional time series obtained from other subject areas to study whether intra-person variability and other data features in longitudinal trajectories can identify different biologic or clinical sub-populations.

## Conclusion

When it is of interest to explore clinical patterns using densely sampled longitudinal data, the hierarchical functional data clustering method can be used for fully data driven unsupervised clustering. The method was applied to the frequently sampled longitudinal Nugent scores to identify different patterns in the natural history of BV in a cohort of Ugandan women. Further risk factor analysis identified demographic and behavioral risk factors associated with persistent BV burden in women. The hierarchical functional data clustering method provides an exploratory data analysis approach for frequent longitudinal data.

## Data Availability

The dataset used analysed during the current study is available from the corresponding author on reasonable request.
